# Pre-pregnancy care for women with pre-gestational diabetes mellitus: a systematic review and meta-analysis

**DOI:** 10.1186/1471-2458-12-792

**Published:** 2012-09-17

**Authors:** Hayfaa A Wahabi, Rasmieh A Alzeidan, Samia A Esmaeil

**Affiliations:** 1 Sheikh Bahmdan Chair of Evidence-based Healthcare and Knowledge Translation, College of Medicine, King Saud University, Riyadh, Saudi Arabia

**Keywords:** Pre-gestational diabetes, Pre-pregnancy care, Congenital malformations, Perinatal mortality

## Abstract

**Background:**

Pre-gestational diabetes mellitus is associated with increased risk for maternal and fetal adverse outcomes. This systematic review was carried out to evaluate the effectiveness and safety of pre-pregnancy care in improving the rate of congenital malformations and perinatal mortality for women with pre-gestational diabetes mellitus.

**Methods:**

We searched the following databases, MEDLINE, EMBASE, WEB OF SCIENCE, Cochrane Library, including the CENTRAL register of controlled trials and CINHAL up to December 2011, without language restriction, for any pre-pregnancy care aiming at health promotion, glycemic control and screening and treatment of diabetes complications in women with type I or type II diabetes mellitus. Study design were trials (randomized and non-randomized), cohort and case–control studies.

**Results:**

Of the 2452 title scanned 54 full papers were retrieved of those 21 studies were included in this review. Twelve cohort studies at low and medium risk of bias, with 3088 women, were included in the meta-analysis. Meta-analysis suggested that pre-pregnancy care is effective in reducing congenital malformation, Risk Ratio (RR) 0.25 (95% CI 0.16-0.37), number needed to treat (NNT) 19 (95% CI 14–24), and perinatal mortality RR 0.34 (95% CI 0.15-0.75), NNT = 46 (95% CI 28–115). Pre-pregnancy care lowers glycosylated hemoglobin A1c (HbA1c) in the first trimester of pregnancy by an average of 1.92% (95% CI −2.05 to −1.79). However women who received pre-pregnancy care were at increased risk of hypoglycemia during the first trimester of pregnancy RR 1.51 (95% CI 1.15-1.99).

**Conclusion:**

Pre-pregnancy care for women with pre-gestational type 1 or type 2 diabetes mellitus is effective in improving rates of congenital malformations, perinatal mortality and in reducing maternal HbA1C in the first trimester of pregnancy. Pre-pregnancy care might cause maternal hypoglycemia in the first trimester of pregnancy.

## Background

Pre-gestational diabetes mellitus (PGDM) and maternal hyperglycemia during the time of organogenesis is a known teratogen with detrimental effects on the fetal heart, renal, musculoskeletal and central nervous systems [1-3]. Population based studies showed that there is a fivefold increase in the rate of cardiovascular malformations, and more than twofold increase in the rate of neural tube defects and urinary tract abnormalities in infants of diabetic mothers when compared to the background population [1,2]. Moreover congenital malformations (GM) are associated with increased risk of stillbirth and perinatal mortality (PM) as they account for almost 50% of all deaths of infants born to mothers with PGDM [4,5].

CM secondary to maternal diabetes can be prevented, in great part, by optimizing maternal health in the pre-pregnancy period. Glycemic control is one of the most important aspects of pre-pregnancy care (PPC) [6]; however other aspects of care such as folic acid supplementation, smoking cessation, screening and treatment of diabetes complications and discontinuing teratogenic medications, are as important for improving maternal and fetal outcomes and might be effective in reducing the rate of CM to the background level [7-9].

The aim of this systematic review is to assess the effectiveness and safety of PPC in improving the CM and perinatal mortality for women with type 1 or type 2 PGDM.

## Methods

### Type of studies

We included in this review randomized trials (including cluster and quasi randomized trials) and cohort and case control studies, comparing the frequency of CM, PM, maternal hypoglycemia in the first trimester and the level of glycosylated hemoglobin A (HbA1C) in diabetic women who received PPC with those who did not receive PPC.

#### Type of participants

Women of reproductive age with type 1 or type 2 PGDM who were not pregnant at the time of intervention.

#### Type of intervention

For the purpose of this review PPC is defined as the following either as sole intervention or in combination

1.Glycemic control by insulin and/or diet aiming at fasting blood glucose ≤5.7 mmol/l or/and postprandial blood glucose ≤7.8 mmol/l and/ or HbA1C ≤7.0%).

2.Women counseling and /or education about diabetes complications during pregnancy, the importance of glycemic control and self monitoring of blood glucose level.

3.Pre-pregnancy screening and treatment of complications of diabetes.

4.The use of contraception until optimization of glycemic control is achieved.

5.Intake of multivitamin or folic acid in the pre-pregnancy period.

#### Type of outcome

Maternal outcomes

1.HbA1C level in the first trimester.

2.Maternal hypoglycemia in the first trimester or any other adverse effect reported by the authors.

Neonatal outcomes

1.CM related to maternal diabetes

2.Perinatal mortality.

#### Exclusion criteria

We excluded from this review reports which are not of comparative design and reports of conference proceedings or abstracts when there is no complete description of the trial or study.

### Search strategy

The search strategy was developed in consultation of an information retrieval specialist. We searched the following databases, MEDLINE (1966-December 2011), EMBASE (1980-December 2011), WEB OF SCIENCE (Science citation index-1970-December 2011), Cochrane Library up to the latest issue 2011, including the CENTRAL register of controlled trials CINHAL (Cumulative Index to Nursing & Allied Health 1982 –December 2011) and Google Scholar. (For full search strategy see Additional file 1: Appendix 1).

We reviewed the reference lists of all relevant studies for any potential study not retrieved by the search strategy. Unpublished reports were not actively sought and there was no language limitation.

### Identification of included studies

All titles and abstracts retrieved by the electronic search were screened independently by the three reviewers, and the studies which clearly did not meet the inclusion criteria, were excluded. Copies of the full text of potentially relevant studies and trials were obtained and their eligibility was assessed independently by two reviews. Differences between reviewers were resolved by discussion or by consulting the third reviewer.

### Data extraction and studies assessment

Two reviews extracted data from the included studies using a designed form. The accuracy of the extracted data was checked by the third reviewer.

The Newcastle Ottawa Scale (NOS) was used for the assessment of cohort, case control studies and non-randomized trials [10]. Risk of bias in each study, was assigned according to the number of items on the NOS judged to be inadequate. We considered low risk of bias when one item is inadequate, medium risk of bias when up to three items are inadequate and high risk of bias when more than three items are inadequate. Risk of bias of the studies included in the review was assessed for each study independently by two reviewers and any disagreement was resolved by discussion with all the reviewers.

Data analysis was carried out with the use of Review Manager Software 5.1.6 (Cochrane Collaboration, Oxford, United Kingdom).

Meta-analysis was performed for studies with similar design and type of intervention, which we assessed to be at medium or low risk of bias using the fixed effect model. Statistical heterogeneity was assessed by visual inspection of forest plots, by performing x^2^ tests (assessing the P value) and by calculating the statistic, which describes the percentage of observed heterogeneity that would not be expected by chance. If the P value was less than 0.10 and I^2^ exceeded 50%, we considered heterogeneity to be substantial. However subgroup analysis was not possible in most of the cases due to the small number of studies. Pooled data were presented as risk ratio (RR) with 95% confidence intervals (95% CI) for dichotomous outcomes and as the means difference with 95% confidence intervals for continuous outcomes.

## Results

**Figure 1:**
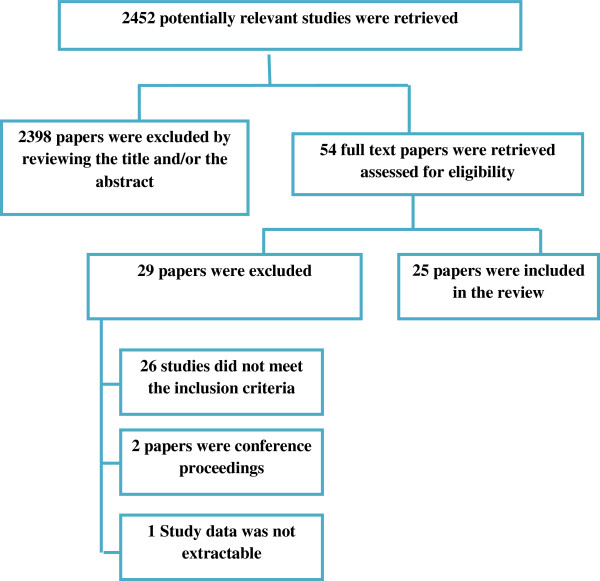
Process of selection of the studies for the systematic review.

The search retrieved 2452 potentially relevant titles of which the full text of 54 relevant reports were reviewed (Figure 1). A total of 25 reports of 21 studies were included in this review [7,8,11-33]. (Three articles described the same cohort study with two interim [15,16] and one final report [17], one study reported the outcomes for the same cohort in two articles [7,27] and two articles report the outcomes of one cohort with one interim [29] and one final report [26]).

Twenty six studies were excluded because they did not meet the inclusion criteria; two reports were of conference proceedings and in one study data were not extractable (Additional file 2: Appendix 2).

Of the included studies, only one was a controlled trial, 13 studies were prospective cohort studies, six studies were retrospective cohort studies and one was a case control study (Tables 1,2,3,4). The included studies were conducted between 1983 and 2010 in Europe and the United States of America, except for one study conducted in Israel [19].

**Table 1:** Characteristics of included prospective cohort studies

**Study/ Year of Publication Reference (country)**	**Participants**	**Intervention**	**Outcome**	**Risk of Bias (Notes)**
**Garcia-Patterson 1997 **[18]** (Spain)**	66 participants with type I and type II who attended the pre-pregnancy clinic and 119 participants with type I and type II diabetes who did not.	PPC included intensive insulin therapy, self-monitoring of blood glucose and dietary advice	The HbA1C was significantly better in the PPC group than for the NPPC group (*p* = 0.01). There was no significant difference between the two groups in rate of congenital malformations.	Medium (The baseline characteristics in relation to the vasculopathy are different. No blinding for the outcome assessment).
**Herman 1999 **[20]** (USA)**	24 women with type I diabetes who attended the pre-pregnancy clinic, and 74 women with type I diabetes who did not attend the pre-pregnancy clinic.	PPC included education, counseling, glycemic control, and assessment of complications of diabetes such as nephropathy and retinopathy	Women who had PPC had significantly lower level of HbA1C at booking and throughout pregnancy. There was no significant difference between the two groups in the frequency of infants with congenital malformations.	High (The study was not designed to assess the clinical outcomes of the pre-pregnancy care but the differences in the socio-demographic features between the groups who attend the pr-pregnancy care and those who did not. The target level for the glycemic control was not clear and the absolute level of Hb A1C at booking and all through pregnancy for the study and the control groups was not mentioned)
**Jaffiol 2000 **[21]** (France)**	21 IDDM attended the pre-conception care and 40 did not attend	PPC included education, glycemic control, self monitoring of blood glucose and contraception	There were significant reductions in the PM and congenital malformations in the PPC group as well as in the level of maternal HbA1C in the 1^st^ trimester compared to the NPPC group.	Low (good report, clear intervention description, the comparative groups received same antenatal intervention. No blinding for outcome assessment)
**Jensen 1986 **[22]** (Denmark)**	9 women with insulin dependent diabetes had pre-pregnancy care and 11 women with insulin dependent diabetic who did not receive pre-pregnancy care.	PPC included continuous insulin infusion initiated 2 months prior to conception.	No significant difference in congenital malformations and HAb1C level, between the two groups	High (small number of study and control group, many differences in the baseline characteristics in the severity of diabetes, 5 of the 11 control women were treated in the diabetic clinic in the hospital before pregnancy so they knew about the importance of glycemic control both groups have the same HA1C levels in early pregnancy)
**Kitzmiller 1991 **[23]** (USA)**	84 women in pre-pregnancy care and 110 women had no pre-pregnancy care.	PPC included glycemic and dietary control education, exercise and contraception.	The frequency of congenital abnormalities in the PPC group was significantly higher than in the NPPC group (*p* <0.05).	Low (good report clear methodology)
**Rosenn 1991 **[24]** (USA)**	28 women in the pre-pregnancy group and 71 in the control group	PPC included dietary advice and glycemic control	HbA1C concentration in the PPC group was lower than in the NPPC group (*p* <0.0008). There were no congenital malformations in either group.	Medium (52% of pre-pregnancy care patients dropped out, no blinding in the assessment of the outcome)
**Temple 2006a **[7]** 2006b **[27]** (UK)**	110 women with type I diabetes attended the pre-pregnancy care clinic and 180 women with type I diabetes did not attend the pre-pregnancy care clinic	PPC included: Glycemic control, folic acid supplementation, smoking cessation, education.	The rate of congenital malformations was lower in PPC group compared to the NPPC group (*p* < 0.065). PM was significantly more in the latter group than the former one (*p* < 0.026)	Low (Baseline characteristics in both groups were similar; the prospective nature of the study ascertained the completeness of the follow up, the completeness of the baseline and the outcome data. Use of appropriate statistical tests such as logistic regression analysis confirmed the association between the pre-pregnancy care and outcomes).
**Willhoite 1993 **[28]** (USA)**	62 women with either type I or type II diabetes who received pre-pregnancy counseling and 123 women witheither type I or type II diabetes who did not receive pre-pregnancy counseling	PPC included counseling by health professional the control group received no counseling.	PPC group had significantly less PM than the NPPC group (OR3.9 CI 1.2-13.9) and insignificantly less congenital malformations (OR 4.2 CI 0.5-29.7)	High (Base line characteristics of the two groups were significantly different in age, duration of diabetes and smoking all are confounding factors for the outcomes. The two groups did not receive the same antenatal intra-partum and postnatal care. The assessor of the congenital malformation was not blinded)
**Boulot 2003 **[31]** (France)**	172 women with either type I or typeII diabetes who received PPC and 260 women with either type I or type II diabetes who did not receive PPC	PPC included education, assessment of diabetes complications glycemic control self monitoring of blood glucose and contraception	PPC group had significantly less PM than the NPPC group, (*p* <0.005) for type 1 diabetics and significantly less congenital malformations, (*p* <0.005) for type 1 diabetics	Low (cases and control were well defined and comparable, selection bias is unlikely as consecutive cases were enrolled, the prospective nature of the study ascertained the completeness of the follow up, the completeness of the baseline and the outcome data)
**Galindo 2006 **[30]** (Spain)**	15 women with pre-gestational diabetes received PPC and 112 women with pre-gestational diabetes did not receive PPC.	PPC included education, glycemic control self monitoring of blood glucose	The frequency of congenital abnormalities in the PPC group was 3/15 compared to 14/112 in the NPPC group.	Low (cases and control were well defined and comparable, selection bias is unlikely as consecutive cases were enrolled, the prospective nature of the study ascertained the completeness of the follow up, the completeness of the baseline and the outcome data)
**Garcia Ingelmo 1998 **[32]** (Spain)**	12 women with pre-gestational diabetes received PPC and 12 women with pre-gestational diabetes did not receive PPC.	PPC glycemic control.	The frequency of congenital abnormalities in the PPC group was 3/12 compared to 2/12 in the NPPC group. HbA1c was significantly lower in the first trimester in the PPC group compared to the NPPC group, (*p* <0.01)	High (Both the study population and the control were not representative of the general diabetic population with frequency of diabetic vascular complications approaching 50%. The PPC components were not defined neither the target blood glucose)
**Murphy 2010 **[8]** (U.K)**	181 participants with type I and type II who received PPC and 499 participants with type I and type II diabetes who did not receive PPC	PPC included: glycemic control, folic acid supplementation, smoking cessation, education.	The frequency of congenital abnormalities in the PPC group was 0.7% compared to 5.6% in the NPPC group (*p* <0.02). The PM in the PPC group was 0.7% which was similar to 2.2% in NPPC (*p* <0.4).	Low (good report clear methodology)
**Evers 2004 **[33]** (Netherland)**	271 women with type I diabetes had planned pregnancy and 152 women did not plan their pregnancy.	PPC had planned pregnancy and folic acid supplementation	The frequency of congenital malformation in PPC group was 11/271 (4.1%) compared to 18/152 (12.2%) in the NPPC group. The mean of HbA1C concentration of the PPC group was significantly lower than the NPPC group.	Medium (confounding factors such as smoking, education level and social class were not examined. The results of HbA1C during the first trimester were not available for 29% of the whole study group)

Key: HbA1c = Glycosylated Hemoglobin A, PPC = Pre-pregnancy Care, NPPC = No Pre-pregnancy Care, OR = Odd Ratio, IDDM = Insulin depended Diabetes Miletus, CI = Confidence Intervals.

**Table 2:** Characteristics of included retrospective cohort studies

**Study/ Year of Publication Reference (country)**	**Participants**	**Intervention**	**Outcome**	**Risk of Bias**
**Dunne 1999 **[14]** (UK)**	47women with IDDM 12 of them attended pre-pregnancy care clinic and 35 women did not.	PPC included assessment of diabetes complications and glycemic control	The PPC group had significantly lower level of HA1C level compared to the NPPC group (*p* < 0.008).	Medium (Due to the audit nature of the report there is no clear description of the intervention, some important confounders were not addressed such as White’s classification and the outcome assessment was not blinded)
			There were no congenital malformations in both groups.	
**Damm 1989 **[13]** (Denmark)**	197 attended PPC and 61 didn’t attend	PPC included: contraception and glycemic control.	The rate of congenital malformations was significantly lower in the PPC group 1.0% than the NPPC group 8.2%, (*p* < 0.01). No significant difference in the level of HA1C during the first trimester between the two group	High (unclear description of the participants, the intervention and the outcome, the data of the pre-pregnancy care were a subset of from different periods of the study)
**Goldman 1986 **[19]** (Israel)**	44 women with type I diabetes attended the pre-pregnancy clinic and 31women with type I diabetes did not attend	PPC included assessment of diabetic complications, Contraception advice, Glycemic control and dietary advice	The NPPC group had 3 infants with congenital abnormalities while the PPC did have any infant with congenital abnormalities	Low (Clear description of participants and intervention, noted confounding factors and well presented results. There was significant difference between the two groups in the diabetes complications before intervention)
**Fuhrmann 1986 **[17]**& 1984 **[16]**& 1983 **[15]** (Germany)**	620 pregnant women with insulin dependent diabetes,183 received pre-pregnancy care 437 women did not	PPC included: short hospitalization every 3 month until conception, education, self monitoring of blood glucose, assessment and treatment of diabetes complications and glycemic control	PPC group had significantly lower rate of congenital malformations 1.1% compared to the NPPC group 7.0% (*p* < 0.01)	Medium (Well described intervention, no blinding for the outcome, no description of the possible confounding factors)
**Rowe 1987 **[25]** (UK)**	21 IDDM 14 received pre-pregnancy care and 7 did not	PPC included	The PPC group had significantly better initial HA1C level (*p* <0.0001).	High (Unclear description of the participants, no description of possible confounding factors, no blinding in assessment of the outcome, small group, high target of HbA1C 5-9%)
		glycemic control, counseling and blood glucose self monitoring		
**Steel 1990 **[26]** & 1982 **[29]** (UK- Scotland)**	143 IDDM women attended the pre-pregnancy care clinic and 96 IDDM women did not attend	PPC included: education, glycemic controlled and contraception	PPC group had lower first trimester HbA1C as compared to NPPC group (*p* < 0.0001) and lower rate of congenital malformations (*p* < 0. 005), maternal hypoglycemia was significantly common in the PPC group than the NPPC (*p* < 0. 001)	Medium (Good description of interventions, contamination of the control who might know about the usefulness of the and the outcome assessment was not blinded)

Key: HbA1C = Glycosylated Hemoglobin A, PPC = Pre-pregnancy Care, NPPC = No Pre-pregnancy Care, OR = Odd Ratio, IDDM = Insulin depended Diabetes Miletus.

**Table 3:** Characteristics of included case–control studies

**Study/ Year of Publication Reference (country)**	**Participants**	**Intervention**	**Outcome**	**Risk of Bias/Notes**
**Correa 2003 **[12]** USA**	Cases were 3278 Infants with congenital malformations related to diabetes. Controls were 3029 infants without congenital malformations. Maternal diabetes and intake of multivitamin were evaluated as a risk factors for congenital malformations	PPC included the use of multivitamin for 3 month before conception	The risk of congenital malformations related to diabetes was limited to infants of f diabetic mothers who had not taken multivitamin (OR 3.39 95% CI 1.79-8.63). Mother who had taken multivitamin had no increase risk of congenital malformations related to diabetes (OR 0.15 95% CI 0.00-1.99)	Medium (clear definition and selection of cases and controls, and outcomes, clearly defined outcome, not clear if the interviewers were blinded to the outcome, recall bias cannot be excluded during the interviews)

Key: PPC = Pre-pregnancy Care, OR = Odd Ratio, CI = Confidence Interval.

**Table 4:** Characteristics of included controlled trials

**Study/ Year of Publication** **Reference (country)**	**Participants**	**Intervention**	**Outcome**	**Risk of Bias/Note**
**Pregnancy outcome in Diabetes control and complication trial research group 1996**[11]** (USA)**	187 had pre-pregnancy intensive insulin therapy and 83 did not.	PPC included glycemic control and dietary advice.	There was one still birth in the PPC group and 3 in the NPPC. Congenital malformations were 5 in the PPC group and 4 in the NPPC group. Mean HbA1C in PPC group was7.4 ± 1.3 compared to 8.8 ± 1.7 in the NPPC group.	High (Unclear report of the outcome, the control group was aware of the importance of glycemic control and was repeatedly advised to change into intensive therapy when planning pregnancy. So intervention was not restricted to the pre-pregnancy group. No specific target level of the blood sugar was stated for the pre-pregnancy group)

Key: HbA1C = Glycosylated Hemoglobin A, PPC = Pre-pregnancy Care, NPPC = No Pre-pregnancy Care.

### Assessment of the methodological quality of the included studies

The cohort studies included in this review (Tables 1 &2) had adequate description of participants including description of some confounding factors such as the frequency of renal and vascular complications of diabetes between the PPC group and the control group. However all studies did not address the effect of the presence of confounding factors on the outcomes except for three reports which used regression analysis to evaluate the effectiveness of the PPC [7,8,27].

In most of the cohort studies blinding of the control group was adequate because they were recruited after pregnancy when they attended for antenatal care, except for two studies [22,26], in which inadequate blinding of the control group cannot be excluded because they were informed about the importance of the PPC and were invited to attend. All participants received the same antenatal and post natal care except for one study [22] where participants were followed up in different health settings.

All cohort studies had adequate follow up for participants except for two studies; in one study 52% of the PPC group were lost to follow up and in the other study 29% of records of HbA1C in the first trimester for the study cohort were missing [24,33]. The assessors of the outcomes were not blinded to the participants’ allocation except in one study [23].

Some of the studies at high risk of bias were initially designed to assess aspects of PPC other than its effectiveness in improving maternal and fetal outcomes, hence the poor methodological design when assessed with the NOS [14,20,28].

PPC in all the cohort studies included control and self monitoring of blood glucose except for one which was designed to examine the effectiveness of pre-pregnancy counseling on fetal and neonatal outcomes [28]. In addition to glycemic control, four studies included screening and treatment of complications of diabetes in the PPC program [14,17,19,20]. Three cohort studies (four reports) had comprehensive PPC program including, control and self monitoring of blood glucose, folic acid supplementation, smoking cessation advice and discontinuation of teratogenic drugs [7,8,27,33].

One case–control study was included in this review [12] (Table 3). It examined the effectiveness of multivitamin supplementation in the pre-pregnancy period in preventing diabetes related CM. The study is at medium risk of bias due to possibility of recall bias during the interview of the mothers and the possibility that interviewers were not blinded to the outcome.

One trial was included in this review [11] (Table 4). The design of the trial was not clear as authors reported it as a randomized trial but the method for randomization was not described. There was no allocation concealment and lack of blinding introduced bias because both groups were aware of the importance of the glycemic control and the complications of diabetes during pregnancy.

Only two studies in this review evaluated maternal hypoglycemia, as an adverse effect of PPC [7,26].

### Outcome of PPC

Similarity of participants, interventions and outcomes in addition to the score of low or medium risk of bias, made meta-analysis possible for 13 cohort studies [7,8,14,17-19,21,23,24,26,30,31,33] with 3411 participants (Tables 1, 2 &5). Both dichotomous and continuous data were pooled but only when standard deviation and similar units were available for continuous data. Studies which were at high risk of bias or of a design other than cohort were excluded from the meta-analysis.

**Table 5:** Pooled estimates effect of pre-pregnancy care

**Dichotomous outcomes of pre-pregnancy care**	**No of studies [reference]**	**Risk Ratio [95% Confidence interval]**
Congenital malformation	13 [7,8,14,17-19,21,23,24,26,30,31,33]	0.25 [0.16-0.37]
PM	6 [7,8,14,18,21,31]	0.34 [0.15-0.75]
Maternal hypoglycemia	2 [7,26]	1.51 [1.15,1.99]
**Continuous outcomes**	**Number of studies references**	**Means difference (95% CI)**
The difference in the level of glycosylated Hemoglobin A1c	5 [7,19,24,26,33]	−2.43 [−2.27 to −2.58]

**Figure 2:**
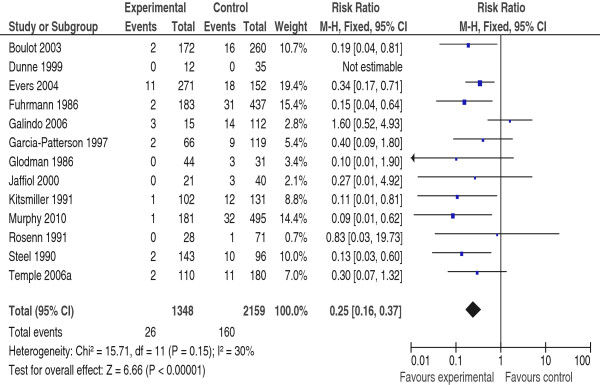
**Risk Ratio for congenital malformation from 13 studies of women with pre-gestational diabetes mellitus who did or did not receive pre-pregnancy care.** PPC (experimental) = the group who received pre-pregnancy care; NPPC (control) = the group who did not received pre-pregnancy care; CI = Confidence intervals.

**Figure 3:**
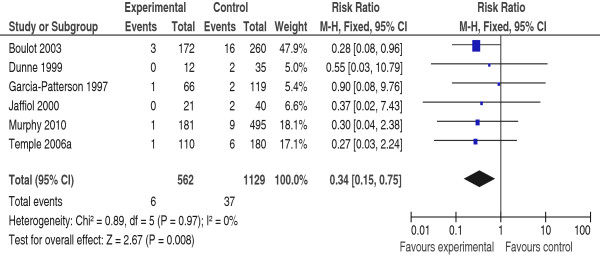
**Risk ratio for (perinatal mortality) from six studies of women with pre-gestational diabetes mellitus who did or did not receive pre-pregnancy care.** PPC (experimental) = the group who received pre-pregnancy care; NPPC (control) = the group who did not received pre-pregnancy care; CI = Confidence intervals.

Meta-analysis suggested that pre-pregnancy care is effective in reducing CM, RR 0.25 (95% CI 0.16-0.37), NNT19 (95% CI 14–24) (Figure 2), and PM RR 0.34 (95% CI 0.15-0.75), NNT = 46 (95% CI 28–115) (Figure 3). There was no evidence of statistical heterogeneity.

**Figure 4:**
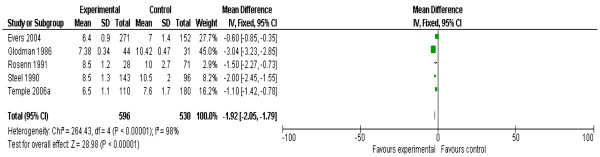
**First trimester mean value of glycosylated hemoglobin A1C from five studies of women with pre-gestational diabetes mellitus who did or did not receive pre-pregnancy care.** PPC (experimental) = the group who received pre-pregnancy care; NPPC (control) = the group who did not received pre-pregnancy care; CI = Confidence intervals.

Meta-analysis of pooled data showed that PPC lowers HbA1C in the first trimester of pregnancy by an average of 1.92% (95% CI 1.79-2.05) and while there is high heterogeneity (I^2^ = 98%) this variation is in the size of the effect rather than the direction (Figure 4).

The use of multivitamins in the pre-pregnancy period as a sole intervention, was evaluated by one case control study [12] and was found not to be effective in reducing the rate of CM, [Odd Ratio (OR) 0.15, 95% CI 0.00-1.99].

Similarly one study, at high risk of bias, evaluated the effectiveness of pre-pregnancy counseling, as a sole intervention, in improving fetal and neonatal outcomes, showed improvement in PM, OR 3.9(95% CL 1.2-13.9) and no improvement in the rate of CM, OR 4.2(95% CL 0.5-29.7) [28].

**Figure 5:**
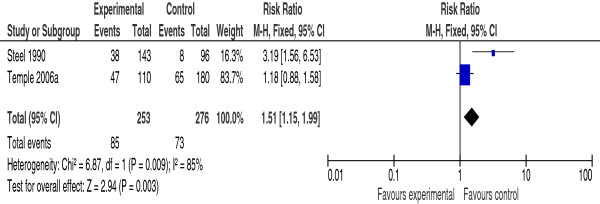
**Risk ratio of maternal hypoglycemia from two studies of women with pre-gestational diabetes mellitus who did or did not receive pre-pregnancy care.** PPC (experimental) = the group who received pre-pregnancy care; NPPC (control) = the group who did not received pre-pregnancy care; CI = Confidence intervals.

Hypoglycemia as an adverse effect of PPC was evaluated by two studies [7,26]. Meta-analysis of the pooled data showed that women who received PPC had significantly more frequent hypoglycemia than those who did not, RR 1.51 (95% CI 1.15-1.99) (Figure 5). However this outcome is associated with marked heterogeneity (I^2^ = 85%).

## Discussion

In this review PPC reduced the rate of CM from 7.4% to 1.9%; a rate similar to that reported for the background population (Figure 2).

Reports from practically implemented PPC programs support our findings of the effectiveness of PPC in improving the rate of CM of infants of mothers with PGDM [8,33]. However, many uncertainties remain about the ability of these programs to reduce the malformation rate to that of the background population, particularly that a small but significant risk of CM remained [33,34]. This observation might be due to many factors, for instance the preferential effect of the PPC on women with type 2 diabetes [8,35]; the influence of other maternal risk factors for CM, such as maternal obesity [36]; and the degree of comprehensiveness; and coverage of services the program provides to prevent malformations beside glycemic control, such as folic acid supplementation and discontinuing teratogenic medication [8].

Population based studies showed a variable increase of 2.5 – 9 folds in the PM rate in diabetic mothers compared to the background population [37]. For women with PGDM 16-28% of PM is due to CM, and an additional 21-41% is due to preterm delivery [38,39]. Since the rates of both complications improve with PPC [9], it is not surprising that the PM in women, who attended PPC, in this review, is reduced by 66% compared to those who did not (Figure 3).

During the period of organogenesis, maternal hyperglycemia is known to be associated with CM [34,40]. Population based studies showed a linear relationship between maternal HbA1C level above 7%, in the first trimester, and both CM and PM [41]. It is estimated that an increase of 1% in the level of HbA1C in the first trimester increases the odds of an adverse pregnancy outcome by 5-6% [42].

The analysis of the pooled data in this review suggested that PPC is effective in reducing the level of HbA1C during the first trimester of pregnancy by 1.9% (Figure 4). However this result is associated with marked statistical heterogeneity (I^2^ = 98%) (Figure 4). The reason for this heterogeneity is the difference in ‘effect size’ produced by the PPC between the included studies. Evers et al. reported a reduction of 8.5% in HbA1C between the intervention and the control groups [33], while Goldman et al. documented a reduction of 29% [19]. Another reason is the difference in the mean levels of HbA1C of the intervention groups (target level), which vary between 6.4% [33] and 8.5% [24,26]. Nevertheless the overall estimate of the effect of PPC in reducing the level of HbA1C is evident by the same direction of effect in all included studies (Figure 4).

In the two studies that evaluated severe maternal hypoglycemia as an adverse effect of PPC [7,26] the pooled data showed an increased risk of hypoglycemia in women who attended the PPC as compared to those who did not (Figure 5). However, this result should be approached with caution due to the marked statistical heterogeneity associated with the meta-analysis (I^2^ = 85%) (Figure 5) and the small number of included studies. The most likely explanation of the statistical heterogeneity is the variable effect size of PPC on maternal hypoglycemia in the two studies. The study by Temple et al. [7] showed no effect of PPC on maternal hypoglycemia, RR 1.18 (95% CI 0.88-1.58), while the study by Steel et al. showed a significant increase in hypoglycemia among women who attended PPC RR 3.19 (95% CI 1.56-6.53) (Figure 5). We believe that the statistical heterogeneity is a reflection of a true clinical heterogeneity. During the 16 year interval between the two studies (Steel et al. 1990, Temple et al. 2006) many innovations in the treatment of diabetes in pregnancy have been developed. Such innovations included patients’ education and counseling, intensive self-monitoring of blood glucose and functional insulin therapy [43]. Although meta-analysis suggested an increased risk of severe hypoglycemia with PPC, we believe this is an unlikely adverse effect with modern treatment and monitoring of diabetes during pregnancy.

In this review some isolated interventions in the pre-pregnancy period were not as effective as a ‘program of interventions’. One case control study addressed the effectiveness of multivitamin supplementation in the pre-pregnancy period, as an isolated intervention, in reducing the rate of CM and showed no effectiveness [12].

One study conducted an economic evaluation of PPC and found that it is associated with considerable savings, and reduced resource utilization [20]. However, population based studies showed that only 34-38% of eligible women receive PPC [3,28]. Hence further studies should focus on how to increase utilization and uptake of PPC and reduction of the rate of unplanned pregnancies among diabetic women.

This systematic review provides a moderate to high level of evidence for the effectiveness of PPC in the improvement of maternal and fetal outcomes [44]. It confirms previous findings by Ray et al. [6]; nevertheless, the strength of our review is based on the comprehensive evaluation of the available evidence on the effectiveness and safety of PPC.

We are aware of the limitations of the observational studies as the main source of evidence and the inherent bias associated with the design; however, randomized controlled trials to assess the effectiveness of PPC are neither ethical nor feasible. Nevertheless the nature of the intervention lent strength to the observational studies by avoiding certain biases known to occur in such study designs. Lack of allocation concealment and blinding of participants were avoided by recruiting the intervention and the control groups at different times during the course of the study (pre-pregnancy period and antenatal period). Additionally, and due to the relatively short duration of the pregnancy, attrition bias was noted in only one study, [24] all other studies had complete follow-up of both groups. The possible bias due to confounding factors such as social class, level of education, subjects’ motivation, smoking, maternal age, obesity, parity and vascular complications of diabetes, was noted by most of the studies but only two studies used the appropriate statistical tests to quantify the effect of the PPC apart from the confounders [7,8]. It is worth noting that all but one of the studies included in this review were conducted in Europe and the United States of America [19], which limits the generalizability of this review.

The review carries important implications for practice and research as it highlights the importance of the integration of PPC in the routine care of diabetic women during their reproductive age. and have practical implication considering the recent report of the CEMCH [3] which showed that CM rate in infants of diabetic mothers in England, Wales and Northern Ireland is twofold, and the PM was nearly fourfold, that of the background population. These findings are also of a paramount importance to many communities in the Middle East [45], North Africa [46] and some communities in Asia [47] where the rate of CM is very high due to many causes including maternal diabetes.

One of the main obstacles to the full implementation of PPC programs is the failure of the target population to utilize provided services [8]. We suggest that more research is needed in methods of encouraging diabetic women to utilize PPC.

## Conclusion

PPC for women with type 1 or type 2 PGDM is effective in improving rates of CM, PM and in reducing maternal HbA1C in the first trimester of pregnancy. PPC might cause maternal hypoglycemia in the first trimester of pregnancy.

## Competing interests

The authors declare that they have no competing interest.

## Authors’ contribution

HW conceived the idea of the review, was responsible for drafting and writing the study protocol and reviewing the search strategy. HW, RZ and SE were responsible for study selection and data extraction. HW and RZ were responsible for quality assessment of studies and data analysis. HW was responsible for writing the final manuscript. HW, RZ and SE reviewed and approved the final manuscript.

## Pre-publication history

The pre-publication history for this paper can be accessed here:

http://www.biomedcentral.com/1471-2458/12/792/prepub

## Supplementary Material

Additional file 1: Appendix 1 Search Strategy. Click here for file

Additional file 2: Appendix 2 Excluded studies. List of studies excluded from the systematic review and the reasons for their exclusion. Click here for file
