# Supplementary material for: Pre-pregnancy care for women with pre-gestational diabetes mellitus: a systematic review and meta-analysis
Source: BMC Public Health. 2012 Sep 17;12:792. doi: 10.1186/1471-2458-12-792 (PMC3575330; doi:10.1186/1471-2458-12-792)
Supplement: Additional file 1: Appendix 1 — Search Strategy. [file 1471-2458-12-792-S1.doc]

**Appendix 1**

**Search Strategy**

**Electronic databases searches.**

We searched the following electronic databases:
-MEDLINE (1966 Dec 2011)
-EMBASE (1980 to Dec 2011)
-WEB OF SCIENCE (Science Citation Index – 1970 to Dec 2011)

-Cochrane Library (up to Dec 2011) – including CENTRAL register of Controlled Trials

-CINHAL (Cumulative Index to Nursing & Allied Health; 1982 to Dec 2011)

**Search Strategy in MEDLINE:**
Exp Diabetes mellitus [Mesh]

1. Pregnancy in diabetics [Mesh]
2. Diabet* ti, ab
3. 1 or 2 or 3
4. Preconception care [Mesh]
5. preconception* AND (service* OR counsel* OR program* OR care OR education* OR clinic*) ti, ab
6. pregestational AND (service* OR counsel* OR program* OR care OR education* OR clinic*) ti, ab
7. periconception AND (service* OR counsel* OR program* OR care OR education* OR clinic*) ti, ab
8. Prepregnancy AND (service* OR counsel* OR program* OR care OR education* OR clinic*) ti, ab
9. Pregnancy in diabetics [Mesh] AND (Hemoglobin A1c ti, ab)
10. Pregnancy in diabetics [Mesh] AND (Hyperglycemia [Mesh] OR blood glucose ti,ab)
11. 5 or 6 or 7 or 8 or 9 or 10 or 11
12. randomized controlled trial [pt]
13. controlled clinical trial [pt]
14. randomized OR randomised ti, ab
15. placebo* ti, ab
16. randomly ti, ab
17. clinical trials as topic [Mesh]
18. intervention* ti, ab
19. evaluat* ti, ab
20. compar* ti, ab
21. Case-control studies [Mesh]
22. Cohort studies [Mesh]
23. retrospective ti, ab
24. prospective ti, ab
25. 13 or 14 or 15 or 16 or 17 or 18 or 19 or 20 or 21 or 22 or 23 or 24 or 25
26. 4 and 12 and 26
