# Supplementary material for: Pre-pregnancy care for women with pre-gestational diabetes mellitus: a systematic review and meta-analysis
Source: BMC Public Health. 2012 Sep 17;12:792. doi: 10.1186/1471-2458-12-792 (PMC3575330; doi:10.1186/1471-2458-12-792)
Supplement: Additional file 2: Appendix 2 — Excluded studies. List of studies excluded from the systematic review and the reasons for their exclusion. [file 1471-2458-12-792-S2.doc]

**Appendix 2**:

**Table of Excluded Studies**

| **Study /Year of Publication** | **Reasons for Exclusion** |
| --- | --- |
| Temple (2011) [1] | Review and recommendation for management |
| Charron-Prochownik (2008) [2] | The trial examines outcomes different from the outcomes of the review. |
| Cousins (1991) [3] | Outcomes of PCC group were not compared to the group who did not receive PCC. |
| Del Rey (2001) [4] | There is no control group to compare outcome with preconception care (PCC) group. |
| Casele (1998) [5] | This is an observational non comparative study. |
| Miller (1981) [6] | The study was on pregnant participants and not preconception group |
| Hod (1995) [7] | The study reports an intervention on a case series with no control group. |
| Jouatte (1999) [8] | This is an observational non comparative study. |
| Rowe (1988) [9] | The study population includes women with impaired glucose tolerance. Outcome for established diabetic participants who received PCC was not reported separately. |
| McElvy (2000) [10] | The study does not compare the outcomes of the PCC group with those of no PCC group. |
| Pearson (2007) [11] | No comparison on the outcome between PCC group and no PCC group. |
| Dicker (1987) [12] | The study compares two types of preconception care rather than PCC to no PCC. |
| Kinsley (2007) [13] | The study does not compare the outcomes of the PCC group with those of no PCC group. |
| Rodgers (1996) [14] | No comparison on the outcome between PCC group and no PCC group. |
| Unger (2001) [15] | Review and recommendation for management. |
| Garcia (1997) [16] | Conference abstract |
| Forde (2006) [17] | Conference proceeding |
| Kitzmiller (1996) [18] | Review and recommendation for management. |
| Mathiesen (2007) [19] | Data for the outcome of PCC not reported. |
| Anwar (2010) [20] | There is no comparative group to compare outcome with preconception care (PCC) group. |
| Avnish Tripathi (2010) [21] | Data are not extractable. |
| Tchobroutsky (1991) [22] | The study compare the outcomes of the type1 diabetic mother before &after changing the obstetric strategies rather than PCC &No PCC |
| Varughese (2006) [23] | The study was on the patients attending general DM clinic, no pregnant participants and no PCC or No PCC groups. |
| Eleanor (2011) [24] | The study compares between Women with DM& healthy women rather than PCC to no PCC. |
| Jennifer (2003) [25] | Review &no compression PCC& no PCC |
| Murphy (2007) [26] | Data for the outcome of PCC& no PCC not reported. |
| Blank (2009) [27] | Review , recommendation for management &components of PCC programs |
| Shahidi(2011) [28] | The study examined outcomes different from the outcomes of the review. |
| Mills(1988 ) [29] | The intervention was after the pregnancy |

Reference List

1. Temple R: **Preconception care for women with diabetes: is it effective and who should provide it?** *Best Pract Res Clin Obstet Gynaecol* 2011, **25:** 3-14.

2. Charron-Prochownik D, Ferons-Hannan M, Sereika S, Becker D: **Randomized efficacy trial of early preconception counseling for diabetic teens (READY-girls).** *Diabetes Care* 2008, **31:** 1327-1330.

3. Cousins L: **The California Diabetes and Pregnancy Programme: a statewide collaborative programme for the pre-conception and prenatal care of diabetic women.** *Baillieres Clin Obstet Gynaecol* 1991, **5:** 443-459.

4. Delgado del RM, Herranz L, Martin VP, Janez M, Juan Lozano GJ, Darias R *et al*.: **[Effect of preconceptional metabolic control in the course of pregnancy in diabetic patients].** *Med Clin (Barc )* 2001, **117:** 45-48.

5. Casele HL, Laifer SA: **Factors influencing preconception control of glycemia in diabetic women.** *Archives of Internal Medicine* 1998, **158:** 1321-1324.

6. Miller E, Hare JW, Cloherty JP, Dunn PJ, Gleason RE, Soeldner JS *et al*.: **Elevated maternal hemoglobin A1c in early pregnancy and major congenital anomalies in infants of diabetic mothers.** *N Engl J Med* 1981, **304:** 1331-1334.

7. Hod M, van Dijk DJ, Karp M, Weintraub N, Rabinerson D, Bar J *et al*.: **Diabetic nephropathy and pregnancy: the effect of ACE inhibitors prior to pregnancy on fetomaternal outcome.** *Nephrol Dial Transplant* 1995, **10:** 2328-2333.

8. Jouatte F, Aitken B, Dufour P, Valat AS, Vamberghe A, Cappoen JP *et al*.: **Diabetes prior to pregnancy: 143 cases.** *Contraception Fertilite Sexualite* 1999, **27:** 845-852.

9. Rowe BR, Barnett AH: **Pre-conception counselling in Asian women with non insulin dependent diabetes and impaired glucose tolerance.** *Diabetes Res* 1988, **8:** 35-38.

10. McElvy SS, Miodovnik M, Rosenn B, Khoury JC, Siddiqi T, Dignan PS *et al*.: **A focused preconceptional and early pregnancy program in women with type 1 diabetes reduces perinatal mortality and malformation rates to general population levels.** *J Matern Fetal Med* 2000, **9:** 14-20.

11. Pearson DWM, Kernaghan D, Lee R, Penney GC: **The relationship between pre-pregnancy care and early pregnancy loss, major congenital anomaly or perinatal death in type I diabetes mellitus.** *BJOG: An International Journal of Obstetrics and Gynaecology* 2007, **114:** 104-107.

12. Dicker D, Feldberg D, Karp M: **Preconceptional diabetes control in insulin-dependent diabetes mellitus patients with continuous subcutaneous insulin infusion therapy.** *Journal of Perinatal Medicine* 1987, **15:** 161-167.

13. Kinsley B: **Achieving better outcomes in pregnancies complicated by type 1 and type 2 diabetes mellitus.** *Clin Ther* 2007, **29 Suppl D:** S153-S160.

14. Rodgers BD, Rodgers DE: **Efficacy of preconception care of diabetic women in a community setting.** *J Reprod Med* 1996, **41:** 422-426.

15. Unger J: **Preconception Management of Women with Type 1 Diabetes.** *The Female Patient* 2001, **26:** 40-46.

16. Garcia A, Corcoy R, Albareda M, Caballero A, Adelantado J, Altirriba O *et al*.: **Diabetic pregnancy: Prepregnancy care and pregnancy outcome.** *Diabetologia* 1997, **40:** 879.

17. Forde R, Connolly C, Murray S, Byrne MM, Firth RG, Kinsley BT: **Benefits of attending a dedicted pre-pregnancy service for women with diabetes mellitus-preliminary findings.** *Irish Journal of Medical Science* 2006, **175:** 21.

18. Kitzmiller JL, Buchanan TA, Kjos S, Combs CA, Ratner RE: **Pre-conception care of diabetes, congenital malformations, and spontaneous abortions.** *Diabetes Care* 1996, **19:** 514-541.

19. Mathiesen ER, Kinsley B, Amiel SA, Heller S, McCance D, Duran S *et al*.: **Maternal glycemic control and hypoglycemia in type 1 diabetic pregnancy: a randomized trial of insulin aspart versus human insulin in 322 pregnant women.** *Diabetes Care* 2007, **30:** 771-776.

20. Anwar A, Salih A, Masson E, Allen B, Wilkinson L, Lindow SW: **The effect of pre-pregnancy counselling for women with pre-gestational diabetes on maternal health status.** *Eur J Obstet Gynecol Reprod Biol* 2011, **155:** 137-139.

21. Tripathi A, Rankin J, Aarvold J, Chandler C, Bell R: **Preconception counseling in women with diabetes: a population-based study in the north of England.** *Diabetes Care* 2010, **33:** 586-588.

22. Tchobroutsky C, Vray MM, Altman JJ: **Risk/benefit ratio of changing late obstetrical strategies in the management of insulin-dependent diabetic pregnancies. A comparison between 1971-1977 and 1978-1985 periods in 389 pregnancies.** *Diabete Metab* 1991, **17:** 287-294.

23. Varughese GI, Chowdhury SR, Warner DP, Barton DM: **Preconception care of women attending adult general diabetes clinics--are we doing enough?** *Diabetes Res Clin Pract* 2007, **76:** 142-145.

24. Schwarz EB, Postlethwaite D, Hung YY, Lantzman E, Armstrong MA, Horberg MA: **Provision of Contraceptive Services to Women with Diabetes Mellitus.** *J Gen Intern Med* 2011.

25. Klinke J, Toth EL: **Preconception care for women with type 1 diabetes.** *Can Fam Physician* 2003, **49:** 769-773.

26. Murphy HR, Temple RC, Roland JM:  **Improving outcomes of pregnancy for women with type 1 and type 2 diabetes.**  *British Journal of Diabetes & Vascular Disease* 2007, **7:** 38-42.

27. Blank H, Wyckoff J:  **Preconception care for women with Diabetes Mellitus.**  *Diabetes in Women :pathophysiology and therapy* 2009, 273-289 .

28. Shahidi S, Aghdak P, Farajzadegan Z, Izadi M, Mohammadi M, Fard MN:  **Reviewing the effectiveness of pre-pregnancy counseling protocol on pregnancy and labor indices.** *IJNMR* 2011, **16**.

29. Mills JL, Knopp RH, simpson JL: **Lack of Relation of Increased Malformation Rates in Infants of Diabetic Mothers To Glycemic Control During Organogenesis.** *N Engl J Med* 1988,  **318:** 671-677.
